# Molecular Dynamics Modeling for the Determination of Elastic Moduli of Polymer–Single-Walled Carbon Nanotube Composites

**DOI:** 10.3390/ijms241411807

**Published:** 2023-07-22

**Authors:** Aigul Shamsieva, Alexander Evseev, Irina Piyanzina, Oleg Nedopekin, Dmitrii Tayurskii

**Affiliations:** Institute of Physics, Kazan Federal University, 420008 Kazan, Russia

**Keywords:** carbon nanotube, polymer composite, molecular dynamics simulation, elastic modulus

## Abstract

The use of carbon nanotubes to improve the mechanical properties of polymers is one of the promising directions in materials science. The addition of single-walled carbon nanotubes (SWCNTs) to a polymer results in significant improvements in its mechanical, electrical, optical, and structural properties. However, the addition of SWCNTs does not always improve the polymer properties. Also, when a certain content of SWCNTs is exceeded, the mechanical properties of the nanocomposite become worse. This article reports the results of computer simulations for predicting the mechanical properties of polymer/single-walled carbon nanotube nanocomposites. The efficiency of reinforcing polymer composites is considered depending on the concentration of carbon nanotubes in the polymer matrix, their size, and structure. The elastic moduli of the nanocomposites are predicted using computer simulations for unit cell tension (0.1%). General trends in the mechanical properties of composites with polypropylene (PP), poly(ethyl methacrylate) (PEMA), polystyrene (PS) matrices, and SWCNTs are shown.

## 1. Introduction

Since the discovery by Iijima in 1991, carbon nanotubes (CNTs) have been one of the most interesting and promising materials for use in various fields of application [1]. Owing to their unique mechanical, optical, and electronic properties, CNTs are of scientific and practical interest for materials science [2,3,4,5]. The main advantages of CNTs are their high rigidity, thermal and electrical conductivity, low density, and high aspect ratio.

One of the possible applications of CNTs is their use as fillers to improve the strength properties of polymeric materials. The choice of polymer and filler is primarily determined by their compatibility. In the case of incompatibility, the resulting composite possesses reduced mechanical properties. A high level of interfacial adhesion makes it possible to distribute the load uniformly with a minimum value of stress concentration and obtain materials with high strength characteristics. Also, of decisive importance is the uniform dispersion of carbon nanotubes in the polymer matrix.

In the development of polymer composite materials, theoretical research is essential to predict the parameters for the use of CNTs. Experimental [6,7,8,9] and theoretical [10,11,12,13] studies have shown that even a small percentage of CNTs in a polymer matrix changes its mechanical properties, such as stiffness and strength. For example, Sokolov et al. reported that adding as small as 1% single-walled carbon nanotubes (SWCNTs) to polypropylene results in a threefold increase in Young’s modulus [6]. They also noted a significant decrease in the slope of modulus vs. SWCNT concentration. Banks-Sills et al. conducted a series of experimental and computerized tests to determine Young’s modulus and Poisson’s ratio for poly(methyl methacrylate) (PMMA) with CNTs. Their experiments showed that an increase in the concentration of CNTs in a polymer leads to a decrease and then to an increase in Young’s modulus and Poisson’s ratio [9]. This behavior can be explained not only by the geometry and orientation of CNTs, but also by the presence of polymer–CNT interfacial interactions. Computer simulation studies also showed that the relationship between interfacial bonding and load transfer between the matrix and fillers cannot be neglected and makes a great influence on the mechanical properties of nanocomposites [12,14,15].

One of the key parameters of a composite is its ability to resist various deformations. Physical quantities that characterize the ability of a material to deform elastically when a force is applied are called elastic moduli. The measurement of stress and strain changes in a composite, including different directions of force, can be performed in various ways, so there are several types of elastic moduli.

The matrix of elastic constants has the following form in general:

$$\left[c\right]=\left[\begin{array}{cccccc} c_{11} & c_{12} & c_{13} & 0 & 0 & 0\\ c_{21} & c_{22} & c_{23} & 0 & 0 & 0\\ c_{31} & c_{32} & c_{33} & 0 & 0 & 0\\ 0 & 0 & 0 & c_{44} & 0 & 0\\ 0 & 0 & 0 & 0 & c_{55} & 0\\ 0 & 0 & 0 & 0 & 0 & c_{66} \end{array}\right]$$

where tensor components give the relationship between the stresses and the resulting deformations for anisotropic systems in different directions.

Young’s modulus, the shear modulus, the bulk modulus, and Poisson’s ratio are determined by the coefficients of the matrix *C_ij_* and the coefficients of the inverse matrix *S_ij_ = C_ij_*^−1^, known as the elastic compliance matrix. It should be taken into account that the experimental values of the elastic moduli of polymers are affected by the methods of sample preparation, the presence of impurities, and equipment. During the modeling, some errors in the values are acceptable since “ideal” composite samples are created, where the interaction of components is described by forcefields. In addition, the general macroscopic rule of mixtures cannot be directly applied to composites with strong interfacial interactions [13]. Also, SWNT dispersion is a critical condition that allows one to control the mechanical properties of the composite [16]. In Ref. [10], the authors present a modeling approach for predicting the effective mechanical properties of polymer nanocomposites modified with SWNTs. Their simulation results with the proposed approach showed good agreement with experimental data for epoxy-SWNT composites. In Ref. [11], the mechanical behavior of CNT/PMMA composite materials subjected to tensile loading is studied using molecular dynamics (MD) simulations. The results of their simulations showed that the Young’s modulus of the PMMA composite reinforced with an infinitely long CNT increases significantly compared to pure PMMA. In addition, they noted that the strength of the CNT/polymer interfacial bond increases with increasing elongation of the CNT fibers and leads to high strength of the composite. In Ref. [12], the results of MD simulations showed that the stronger the interfacial interaction between the CNT and the polymer matrix, the lower the Young’s modulus as a result of the compressibility of the interfacial region. It was also concluded that the presence of an optimal interfacial interaction between a polymer and CNTs is one of the key factors in improving the mechanical properties of nanocomposites. The modification of CNTs can help improve the interaction between CNTs and the polymer matrix. In Ref. [17], it was noted that NH_2_-multi-walled carbon nanotubes (MWCNTs) modified with multifunctional initiators of surface-initiated radical polymerization (MPIs) are easily dispersed in a thermoplastic elastomer matrix and improve the mechanical properties of nanocomposites in comparison with non-modified fillers.

One of the key characteristics of a polymer composite material is the glass transition temperature (*T_g_*), at which the polymer passes from a liquid or highly elastic state to a glassy one. This parameter allows one to determine the application area of the composite material. Experiments show that the introduction of nanofillers affects the crystalline behavior and structure of the polypropylene matrix; in particular, the mechanisms of heterogeneous nucleation are accelerated [18]. It was also found that the crystallinity of the polymer increases with the addition of CNTs [19,20]. For example, Sterzyński et al. reported that the glass transition temperature of a poly(vinyl chloride) (PVC) composite increases with the MWCNT content using differential scanning calorimetry (DSC) and dynamic mechanical thermal analysis (DMTA) methods [21]. The simulation results in Ref. [22] showed that the addition of CNTs to epoxy resin leads to an increase in the glass transition temperature. By means of MD simulations, Wei at al. [23] also showed that the addition of CNTs to a polymer leads to an increase in the glass transition temperature. In Ref. [24], the authors also concluded by means of simulations that the addition of CNTs to polyethylene leads to an increase in *T_g_.*

To sum up, many experimental and calculated works have been published to date considering composites of polymers and CNTs. And most works based on computer simulations describe experimental works quite well. However, their implementation is complicated. This is related first to the significant computational resources required. In addition, there is no unified methodology for the description of polymer-SWNT composites. Consequently, the purpose of this work is to present a molecular dynamics procedure for modeling the polymer-SWNT composite mechanical properties and to evaluate its glass transition temperature as well as to predict the behavior of the elastic moduli in polymer-SWNT composites using computer simulations in a wide range of SWNT concentrations. Composites with low (2–10%) and high (19–70%) SWCNTs are considered within the present research. The mass fraction of nanotubes in the composite is calculated using the following formula: $\omega_{cnt}=\frac{m_{cnt}}{m_{pol}+m_{cnt}},\;$ where *m_cnt_* is the mass of the nanotube and *m_pol_* is the mass of the polymer chain. Also, a comparison with experimental and theoretical data is performed where available. Overall, the influence of the concentration, diameter, and type of SWCNTs on the mechanical properties of polypropylene, poly(ethyl methacrylate), and polystyrene (PS) is thoroughly investigated. These materials are thermoplastic polymers, which are widely used in the manufacture of products for aviation, the medical industry, construction, instrumentation, and household products. The addition of CNTs would help to increase the strength of these materials and extend their service life.

## 2. Results and Discussion

### 2.1. Effect of Nanotube Concentration

#### 2.1.1. Determination of Elastic Moduli for Polymer-SWCNT

**Figure 1:**
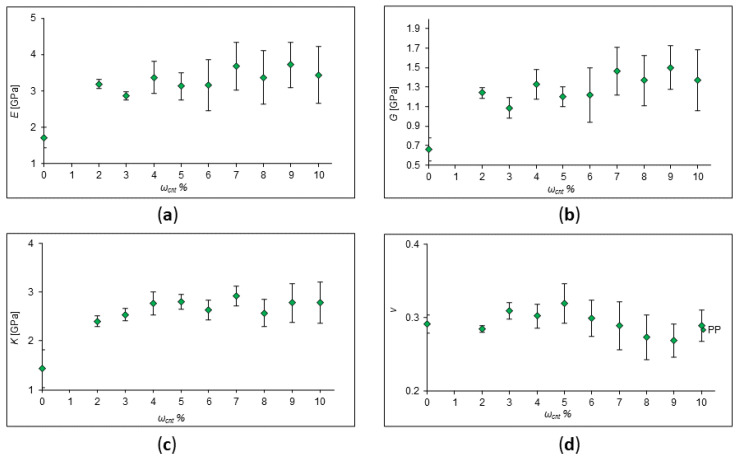
Calculated values of elastic moduli of pure PP and PP with SWCNT concentration of 2–10%. (**a**) Young’s modulus behavior; (**b**) shear modulus behavior; (**c**) behavior of the bulk modulus; (**d**) behavior of Poisson’s Ratio.

In the first stage, the polypropylene (PP) with the addition of zig-zag CNTs was chosen for the impact of SWCNT concentration on the mechanical properties’ investigation. PP is thermoplastic, capable of reversibly transforming into a highly elastic or viscous state when heated. The molecular weight of the considered PP for different SWCNT concentrations is given in Table 1 along with the number of monomer units. The elastic moduli of polypropylene (PP) with the addition of one zig-zag CNT, with chirality indices of (9;0) and a length of 18 Å, were calculated and are plotted in Figure 1, where values of Young’s modulus (*E*), shear modulus (*G*), bulk modulus (*K*), and Poisson’s ratio (*ν*) are presented. It is clearly seen from the plots that a possible nonlinear behavior of the polymer upon addition of CNTs takes place.

Let us discuss the obtained behavior. As a result of these calculations, we see that the ability of the material to resist deformation increases. However, this occurs in a non-linear manner. The same behavior was observed in many experiments [6,9,25,26,27]. This can be explained by the fact that there is an interfacial interaction between the nanotube and the polymer. The arrangement of nanotubes also plays an important role: the more ordered it is, the more rigid the composite in the direction of most nanotubes’ arrangement. In Ref. [25], the results obtained from differential scanning calorimetry curves showed that the addition of SWCNTs to isotactic polypropylene in small amounts (less than 1 wt%) leads to an increase in Young’s modulus and tensile strength. At a concentration of 1%, both stiffness and strength decrease significantly. Also, the results of tensile tests have shown that the inclusion of nanotubes in an amount of less than 1% by weight increases the tensile strength due to the strong interfacial interaction relative to the un-reinforced polymer. In Ref. [27], the authors reported that CNTs significantly affect the mechanical properties of PP/CNT, in particular, the modulus of elasticity and strain at failure. They claimed that the elastic modulus slightly increases at a low CNT content (1–5%). A further increase in the content of CNTs leads to a decrease in the elastic modulus, but it is still higher than that of pure PP. However, elongation at break sharply decreases with increasing CNT content, in agreement with Ref. [27] and other works. This is obvious since the nanotubes make the polymer more rigid and inelastic. Tjong et al. studied polypropylene nanocomposites reinforced with 0.1, 0.3, 0.5, and 1.0 wt% MWCNTs [20]. In their work, dynamical mechanical analysis (DMA) showed that the elastic modulus and thermal bending temperature of a PP/MWCNT nanocomposite are improved by about 33% by adding only 0.3–0.5 wt% of MWCNTs.

**Figure 2:**
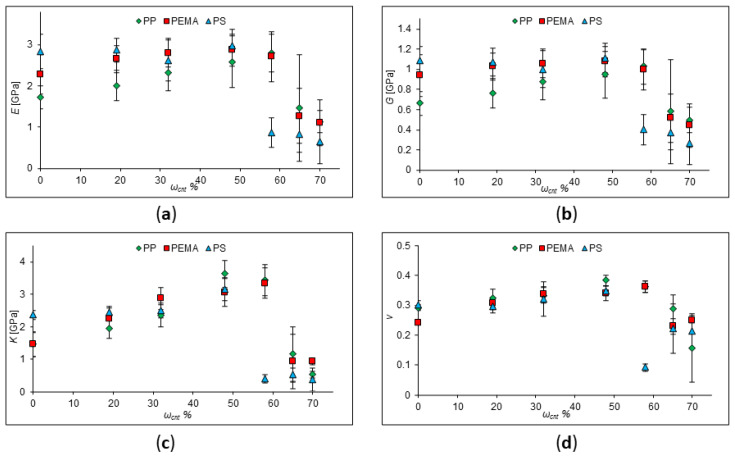
Calculated values of elastic moduli of pure PP, PEMA, and PS, and PP, PEMA, and PS with SWCNT concentration of 19–70%. (**a**) Young’s modulus behavior; (**b**) shear modulus behavior; (**c**) behavior of the bulk modulus; (**d**) behavior of Poisson’s ratio.

In the next stage, the change in the elastic moduli was determined at high (more than 19%) SWCNT concentrations. The elastic moduli of PP, PEMA, and PS with the addition of CNTs, chirality indices of (9;0), and a length of 18 Å were investigated (Figure 2). Parameters of SWCNTs and polymer chains are given in Table 2. For these cells, the values of Young’s modulus, shear modulus, bulk modulus, and Poisson’s ratio were obtained. According to the data obtained for polypropylene, it can be seen that in the composite, the values of Young’s modulus, shear modulus, and bulk modulus decrease, but they are greater than for the unfilled polymer as long as their concentration is less than 60%. The values of all elastic moduli drop sharply with a further increase in the SWCNT concentration. This may be due to the fact that an insufficient amount of polymer cannot hold the SWCNT together by van der Waals forces, and the composite is destroyed at the same values of the mechanical stress.

To sum up, we have shown that at low concentrations (less than 10%), nanotubes can increase the elastic moduli of the polymer. However, this phenomenon will be maintained only within certain limits. Thus, for SWCNTs (with a high modulus of elasticity and low strain to rupture), direct proportionality is not maintained at high degrees of reinforcement, since the matrix (binder) drags the fibers along during deformation, which leads to the destruction of the composite. At high degrees of reinforcement, the lack of a binder to fill the interfiber space above the critical value leads to a violation of the solidity of the composite and, accordingly, the appearance of uneven stresses in it; therefore, destruction occurs at lower values of mechanical stresses than for monolithic samples.

#### 2.1.2. Determination of Glass Transition Temperature for Polypropylene

**Figure 3:**
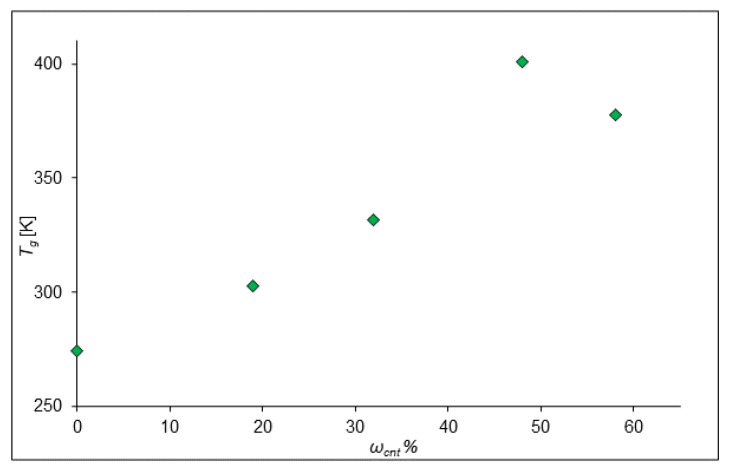
Dependence of the glass transition temperature on the mass fraction of CNTs with polypropylene matrix.

In the next stage, for these configurations, the glass transition temperatures were calculated. The results are presented in Figure 3 where the dependence of the glass transition temperature of the composite on the mass fraction of SWCNTs is shown. It was found that the incorporation of nanotubes leads to an increase in the glass transition temperature (*T_g_*) of the composite, since the mobility of the polymer chain decreases, and the amorphous polymer becomes more rigid.

The obtained values show the dependence of the glass transition temperature of the composite on the mass fraction of SWCNTs. When CNTs are added to the composite, the ordered arrangement of some individual parts increases, which leads to an increase in the glass transition temperature. So, this agrees with the conclusions made by Tjong et al. [20]. They found that adding even a small amount of MWCNTs to PP results in a shift in the glass transition temperature toward higher temperatures. However, the addition of CNTs can also lead to a decrease in the glass transition temperature. So, in an article by Castillo et al., composites based on polycarbonate with MWCNTs demonstrated a glass transition temperature lower than that of pure polycarbonate [28]. They reported that MWCNTs reduced the amount of polymeric material involved in the glass transition of composites, which affected *T_g_*.

Thus, it has been demonstrated that the introduction of fillers affects the crystalline behavior and structure of the polypropylene matrix. The microstructure and distribution of CNTs in the polymer matrix are very important for obtaining a material with high-strength characteristics. However, due to the weak interaction at the CNT-polymer interface in composites, as well as defects in the structure, decreases in the mechanical properties and thermal stability of the composite are possible; as a result of this, the monolithicity of the composite is violated.

### 2.2. Effect of Nanotube Size

**Figure 4:**
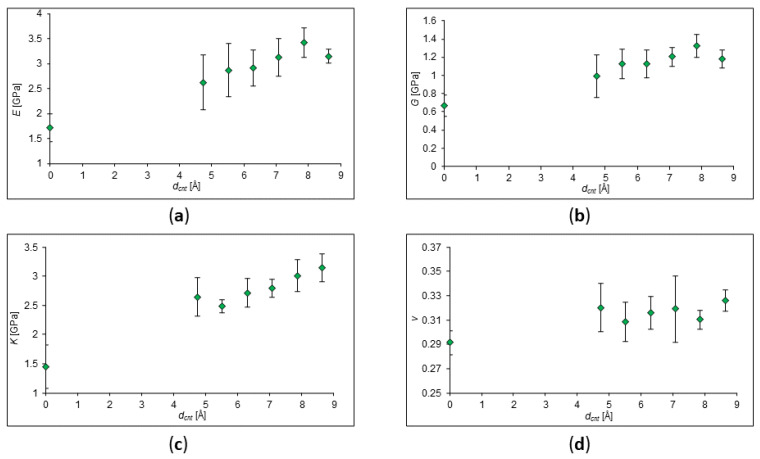
Dependencies of elastic moduli on different SWCNT diameters in the composite with PP matrix. SWCNT concentration in PP is 5%. (**a**) Young’s modulus behavior; (**b**) shear modulus behavior; (**c**) behavior of the bulk modulus; (**d**) behavior of Poisson’s Ratio.

In the present chapter, the effect of size of the filler on the strength characteristics of the composite is investigated. To maintain plasticity and strength, the filler must be “shrouded” in a polymer matrix. Parameters of SWCNTs and polymer chains are given in Table 3. The dependences of the elastic moduli of polypropylene on the addition of zig-zag CNTs were obtained and are demonstrated in Figure 4. The concentration of CNTs in polypropylene is the same everywhere and equals about 5%. It was obtained that with an increase in the radius of nanotubes, the values of the elastic moduli can also increase.

It can be seen from the obtained values that an increase in the SWCNT diameter in polypropylene leads to an increase in Young’s modulus, bulk modulus, and shear modulus. Indeed, Nam et al. for the CNT/epoxy resin composite stated that it is better to use smaller-diameter CNTs to achieve high mechanical properties [29]. In addition, our results agree with another study of Hegde et al. where a linear increase in Young’s modulus with CNT diameter in poly(ether imide) [30] was established. However, in the article by Zhu et al., it was demonstrated that structural changes in the MWCNT/bismaleimide composite significantly affected the phonon and electron transport in the composite structure, but an increase in the length and diameter of CNTs did not lead to a noticeable change in the mechanical properties of the composites [31]. Indeed, some characteristics do not demonstrate a significant and noticeable change in our calculations. However, our calculations agree with other computational research, for instance, with Ref. [32], where it was claimed that a change in the CNT size can also lead to a deterioration in the strength characteristics of the composite.

**Figure 5:**
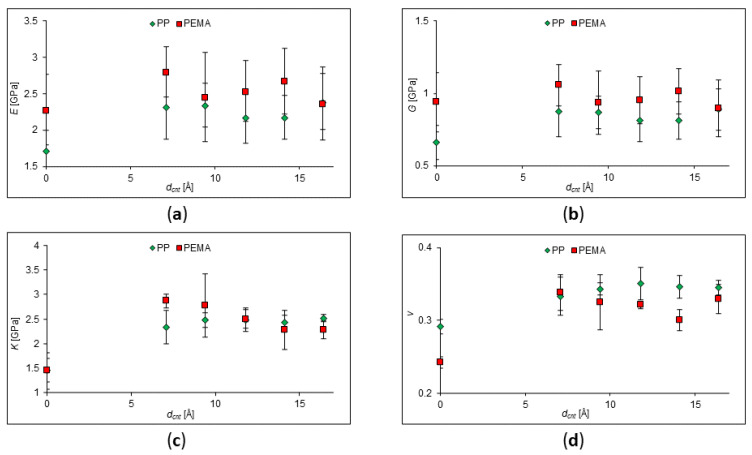
Dependencies of elastic moduli on different SWCNT diameters in the composite with PP and PEMA matrix. SWCNT concentration is 30%. (**a**) Young’s modulus behavior; (**b**) shear modulus behavior; (**c**) behavior of the bulk modulus; (**d**) behavior of Poisson’s Ratio.

The dependences of the elastic moduli of polypropylene and poly(ethyl methacrylate) with the addition of zig-zag CNTs were obtained and are presented in Figure 5. The SWCNT concentration in this study is 30%. The parameters of SWCNTs and polymer chains are given in Table 4. The obtained values indicate that the elastic moduli of the composites depend insignificantly on the diameter of carbon nanotubes at high concentrations. It can also be assumed that there is a critical value of the CNT diameter, after which the strength characteristics of the composite would decrease.

### 2.3. Effect of Nanotube Types

**Figure 6:**
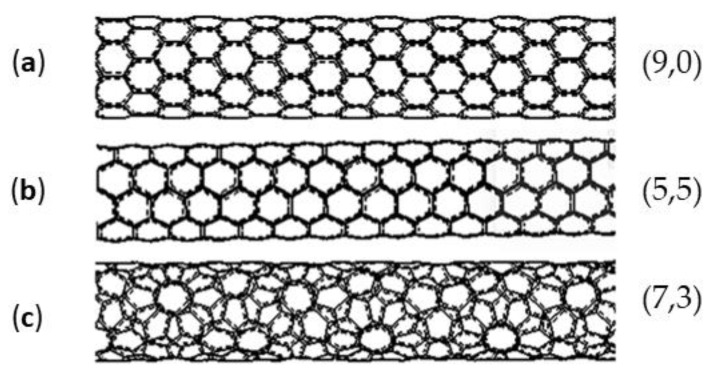
SWNT types used in calculations. (**a**) Zig-zag; (**b**) chair; (**c**) chiral. The chirality indices of CNTs (m, n) are shown in the figure. In this study, they are selected so that SWNTs have approximately the same diameter of 7 Å.

**Figure 7:**
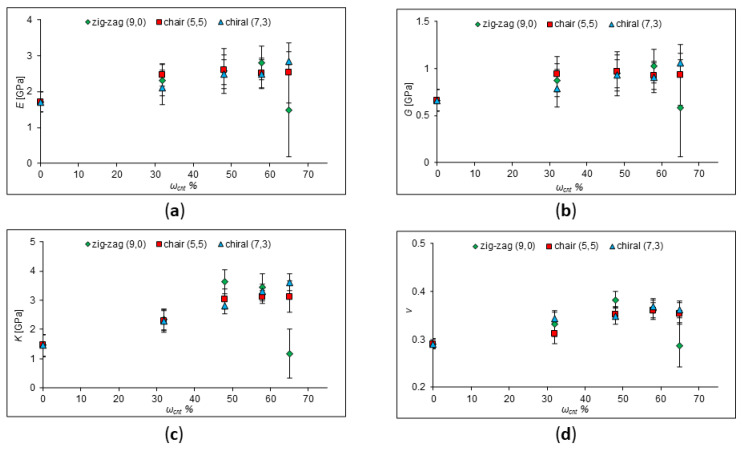
The calculated values of the elastic moduli of pure PP and PP with the addition of zig-zag, armchair, and chiral SWCNTs at a concentration of 32–65%. (**a**) Young’s modulus behavior; (**b**) shear modulus behavior; (**c**) behavior of the bulk modulus; (**d**) behavior of Poisson’s Ratio.

It is well known that a carbon nanotube is an allotropic modification of carbon, which is a hollow cylindrical structure. The nanotube has its own chirality and is characterized by two integers (m, n), which determine the mutual location of the hexagonal grids. The structure of a CNT significantly affects its properties, which can also affect the properties of composites. According to calculations of Young’s modulus, it was found that CNTs with an “armchair” and “zig-zag” configuration are stronger (*E* = 935.8 GPa and *E* = 935.3 GPa, respectively) than tubes with the same diameter but chiral configuration (*E* = 918.3 GPa) [33]. In the article of El-Borgi et al., the chirality of CNTs significantly affected the elastic behavior of nanocomposites [34]. Calculations by Aluko et al. of epoxy/CNT nanocomposites revealed that chirality affects the mechanical characteristics of nanocomposites [35]. Gupta et al. investigated the effect of chirality on the properties of the composite using a multiscale simulation method and concluded that zig-zag nanotubes provide better reinforcement of the composite compared to armchair nanotubes [36]. Kumar et al. also investigated the effect of CNT chirality in a composite using computer simulations. Their results showed a strong effect of chirality at CNT volume fractions up to 0.06 [37]. Therefore, it is interesting to study the influence of the type of nanotube on the strength characteristics of polymer composites by means of molecular dynamic simulations. For this purpose, the effect of adding chair, zig-zag, and chiral nanotubes (chirality indices of nanotubes (5,5), (9,0), and (7,3), respectively, as presented in Figure 6) on the strength characteristics of a polymer composite with a polypropylene matrix was determined and is plotted in Figure 7. The CNT diameters are the same and equal to 7 Å.

According to the dependences obtained, Young’s modulus, the shear modulus, Poisson’s ratio, and the bulk modulus do not differ much for different types of nanotubes at a high concentration of SCNTs. Therefore, it can be assumed that the type of CNTs affects the strength characteristics of the composite insignificantly, and the dispersion of CNTs, their concentration, and size have a much greater effect.

## 3. Materials and Methods

In this work, classical molecular dynamics (MD) simulations were utilized, employing all-atom force fields. To perform the simulations and determine the physical characteristics of the polymer composites, we used the MedeA® Software Environment [38], which incorporates the LAMMPS code developed by Plimpton [39]. Classical MD tracks the evolution of a system of interacting atoms or particles by integrating their equations of motion. However, this approach is not suitable for situations where quantum effects need to be considered or for time scales shorter than the relaxation time of physical quantities. To describe the interactions between atoms within the classical MD simulations, force fields are utilized. In our study, we selected the polymer-matched force field called PCFF+ [40]. The PCFF+ force field is specifically tailored for polymers, and organic and inorganic materials, and can also be used to calculate mechanical properties.

From a computational point of view, it is difficult to calculate the strength of large polymer systems; therefore, in this work, a representative elementary volume (RVE) of a polymer composite was simulated with periodic boundary conditions. According to the type of molecular structure, the polymers in this study were atactic. Nanotubes were in an arbitrary position for 30 simulated structures. All the obtained values of the elastic moduli were averaged over the generated configurations.

It is also difficult to calculate the strength of polymer composite systems for large deformations, since the probability of irreversible/plastic deformations increases. In addition, small deformations can increase the uncertainty in the calculations due to numerical errors. That is why the present study focuses only on the calculation of elastic moduli at low strain. Mechanical properties were calculated using a strain of 0.001 (0.1%). Thus, in this work, the general trends in the mechanical properties of composites versus CNTs composition are presented.

First, a polymer unit was created, then a polymer chain and a nanotube were built, from which a representative elementary volume (RVE) of a polymer composite was created at a temperature of 298.2 K and a polymer density of 0.9 g/cm^3^. For each study, 30 configurations were randomly constructed, and the obtained values were all averaged.

The simulated cells were all equilibrated and deformed. The equilibration was carried out using NVT and NPT ensembles. The Ewald summation method was used to calculate long-range interactions, and the cutoff radius was 9.5 Å [41]. Temperature and pressure were controlled using a thermostat and barostat proposed by Berendsen [42]. After equilibration, the energy was minimized by the conjugate gradient method [43,44]. The value of deformation used for structural analysis was 0.1%.

**Figure 8:**
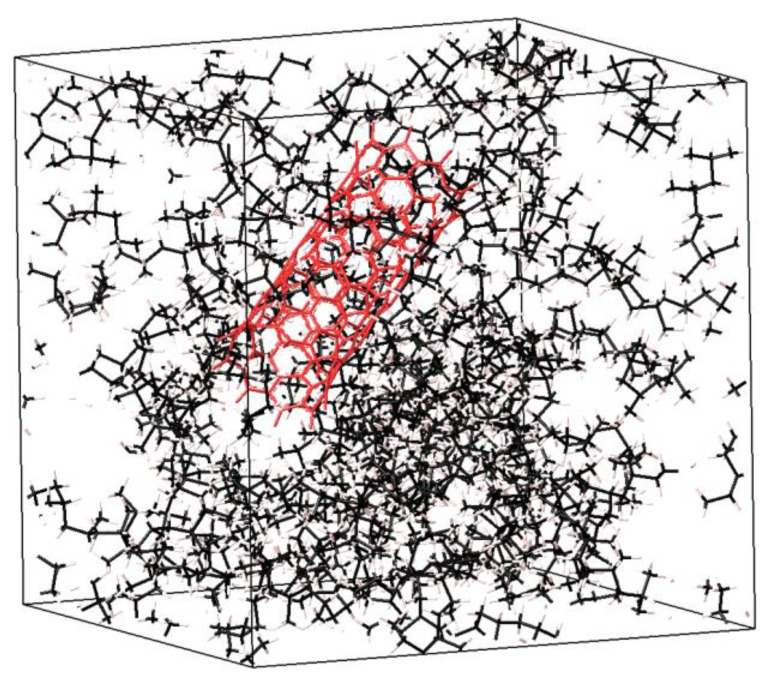
View of the simulated cell used in the present research. In this case, a cell of polypropylene is shown, consisting of 1 chain with a degree of polymerization N = 100. The molecular weight of polypropylene is Mn = 4210 g·mol^−1^. The nanotube has a chirality index of (9;0) and length of 18 Å. The nanotube (shown in red color) is oriented in a random direction.

The calculations were carried out in the following order: First, the variables for the initial and final temperature, integration step, and pressure were set. Then, a series of ensembles was applied in the following order: relaxation in the NVT ensemble at a temperature of 298 K for 5 ps with a step of 0.1 fs. Next, in the NVT ensemble, the cell temperature was varied from 500 K to 298 K in 5 ps with a step of 1 fs. Then, in the NPT ensemble, the pressure was changed from 1000 atm to 1 atm for 50 ps with a step of 1 fs. In the NVT ensemble, the cell temperature varied from 500 K to 298 K in 50 ps with a step of 1 fs. In the NTP ensemble, relaxation was carried out at a temperature of 298 K for 100 ps. The cell then relaxed at a temperature of 298 K for 10 ps with an integration step of 1 fs. Then, the deformation of the polymer structure was set. With small deformations, about 90% mechanically stable structures can be obtained (with the stiffness matrix being positive and non-negative eigenvalues) as based on the mechanical properties’ calculation results. Thus, a matrix of elastic constants was calculated for the chosen configurations, and the elastic moduli and Poisson’s ratio were determined. The elastic moduli were calculated according to Voigt and Reuss, which represent the averaging of the elasticity matrix (*C_ij_*) and compliance matrix (*S_ij_*) and correspond to the extreme upper and lower bounds [45,46]. To determine the final value of the elastic moduli, the Hill approximation was used, which is the arithmetic mean of the boundaries according to Voigt and Reuss [47]. According to the type of molecular structure, the polymers in this study were all atactic, i.e., stereoisomeric configurations at all centers of the main chain were arranged randomly. The nanotubes in the RVE were arranged randomly as shown in Figure 8. In addition, each modulus of elasticity was averaged over the values of 30 cells.

The MD simulations were carried out first in the NVT ensemble at initial T = 298 K and final T = 300 K with a 1 fs integration time step and in the Berendsen thermostat. A relaxation annealing was carried out as follows: the system was first heated to high temperature and then slowly cooled down. This process was carried out using the NPT ensemble with a 1 fs integration time step. Also, the Berendsen thermostat and barostat were considered to keep the system at the prescribed temperatures and pressures. The nonbonded interactions were computed using the Ewald summation method and a nonbond cutoff equal to 9.5 Å. The simulated dilatometry was used with a heating rate of 3 × 10^13^ K/min, i.e., the system was first heated up by 50 K steps with 100 ps of duration; and with a cooling rate of 7.5 × 10^12^ K/min, i.e., the system was then cooled down by 25 K steps with 200 ps of duration. For each temperature, the specific volume, i.e., the inverse density, was reported. Then, averaging over all configurations was performed. Linear fitting was applied for the first and second part of the obtained sequence and the break of the slope yielded T_g_.

## 4. Conclusions

In this work, we studied the effect of nanotubes on the elastic moduli of polymer composite materials using atomistic simulation. The tendencies of the mechanical properties of composites depending on the concentration of SWCNTs were determined. In particular, it was evaluated that at low concentrations of SWCNTs, the value of the strength characteristics increased. However, after reaching critical mass, their values decreased sharply. In addition, it was determined that with an increase in the concentration of nanotubes, the glass transition temperature of the composite increased. However, this dependence was observed only until the critical value of the filler content in the composite was reached. It is a well-known fact that the agglomeration of the filler leads to a decrease in the strength of the composite, as was also shown in this study. We found that the values of the elastic moduli of the polymer composite depended on the SWCNT diameter. In particular, the addition of large-diameter SWCNTs can lead to a decrease in the strength characteristics of the composite. Finally, we found that the type of SWCNT structure did not exhibit a significant effect on the strength of the polymer composite at a high concentration of SWCNTs.

## Figures and Tables

**Table 1:** Dependence of SWCNT concentration on polymer molecular weight. For each SWCNT concentration, a specific molecular weight Mn of polypropylene in the cell is selected as listed in the corresponding column. Each cell is represented by one chain consisting of N monomer units. One zig-zag nanotube with a chirality index of (9,0) and length of 18 Å is used.

SWCNT Concentration	Mn (g·mol^−1^)	N
pure	4210	100
2%	96,232	2287
3%	63,500	1509
4%	47,136	1120
5%	37,315	887
6%	30,768	731
7%	26,092	620
8%	22,585	537
9%	19,857	472
10%	17,675	420

**Table 2:** Dependence of SWCNT concentration on polymer molecular weight. In this study, zig-zag nanotubes with a chirality index of (9;0) and length of 18 Å are used. For each SWCNT concentration, a specific molecular weight Mn of PP, PEMA, and PS in the cell is selected. The number of SWCNTs in a cell is n. Each cell is represented by one chain consisting of N monomer units.

SWCNT Concentration	n	Mn (g·mol^−1^) of PP	N of PP	Mn (g·mol^−1^) of PEMA	N of PEMA	Mn (g·mol^−1^) of PS	N of PS
pure	0	4210	100	4225	37	4168	40
19%	1	8420	200	8450	74	8336	80
32%	1	4210	100	4225	37	4168	40
48%	2	4210	100	4225	37	4168	40
58%	3	4210	100	4225	37	4168	40
65%	4	4210	100	4225	37	4168	40
70%	5	4210	100	4225	37	4168	40

**Table 3:** Dependence of SWCNT diameter on polymer molecular weight. The concentration of CNTs in polypropylene is about 5%. For each SWCNT diameter, a specific molecular weight of Mn PP in the cell is chosen. The number of SWCNTs in a cell is one, (n,m) are chirality indices, l is the SWCNT length. Each cell is represented by one chain consisting of N monomer units.

SWCNT Diameter, (Å)	(n,m)	l, (Å)	Mn (g·mol^−1^)	N
-	-	-	4210	100
4.7	(6,0)	12	16,661	396
5.5	(7,0)	14	22,633	538
6.3	(8,0)	16	29,517	702
7.1	(9,0)	18	37,314	887
7.8	(10,0)	20	46,025	1094
8.6	(11,0)	22	55,648	1323

**Table 4:** Dependence of SWCNT diameter on polymer molecular weight. The concentration of CNTs in polymers is about 30%. For each SWCNT diameter, a specific molecular weight of Mn PP and PEMA in the cell is chosen. The number of SWCNTs in a cell is one, (n,m) are chirality indices, l is the SWCNT length. Each cell is represented by one chain consisting of N monomer units.

SWCNT Diameter, [Å]	(n,m)	l, [Å]	Mn [g·mol^−1^] of PP	N of PP	Mn [g·mol^−1^] of PEMA	N of PEMA
-	-	-	4210	100	4225	37
7.1	(9,0)	18	4210	100	4225	37
9.4	(12,0)	18	4210	100	4225	37
11.8	(15,0)	18	4210	100	4225	37
14.1	(18,0)	18	4210	100	4225	37
16.4	(21,0)	18	4210	100	4225	37

## Data Availability

The data presented in this study are available on request from the corresponding author.
